# From Fast Oscillations to Circadian Rhythms: Coupling at Multiscale Frequency Bands in the Rodent Subcortical Visual System

**DOI:** 10.3389/fphys.2021.738229

**Published:** 2021-11-26

**Authors:** Lukasz Chrobok, Mino D. C. Belle, Jihwan Myung

**Affiliations:** ^1^Department of Neurophysiology and Chronobiology, Institute of Zoology and Biomedical Research, Jagiellonian University in Krakow, Krakow, Poland; ^2^Institute of Clinical and Biomedical Sciences, University of Exeter Medical School, University of Exeter, Exeter, United Kingdom; ^3^Graduate Institute of Mind, Brain, and Consciousness, Taipei Medical University, Taipei, Taiwan; ^4^Brain and Consciousness Research Centre, Taipei Medical University-Shuang Ho Hospital, Ministry of Health and Welfare, New Taipei City, Taiwan

**Keywords:** subcortical visual system, multiscale frequency, circadian clock, gamma oscillation, infra-slow

## Abstract

The subcortical visual system (SVS) is a unique collection of brain structures localised in the thalamus, hypothalamus and midbrain. The SVS receives ambient light inputs from retinal ganglion cells and integrates this signal with internal homeostatic demands to influence physiology. During this processing, a multitude of oscillatory frequency bands coalesces, with some originating from the retinas, while others are intrinsically generated in the SVS. Collectively, these rhythms are further modulated by the day and night cycle. The multiplexing of these diverse frequency bands (from circadian to infra-slow and gamma oscillations) makes the SVS an interesting system to study coupling at multiscale frequencies. We review the functional organisation of the SVS, and the various frequencies generated and processed by its neurons. We propose a perspective on how these different frequency bands couple with one another to synchronise the activity of the SVS to control physiology and behaviour.

## Introduction

**Figure 1:**
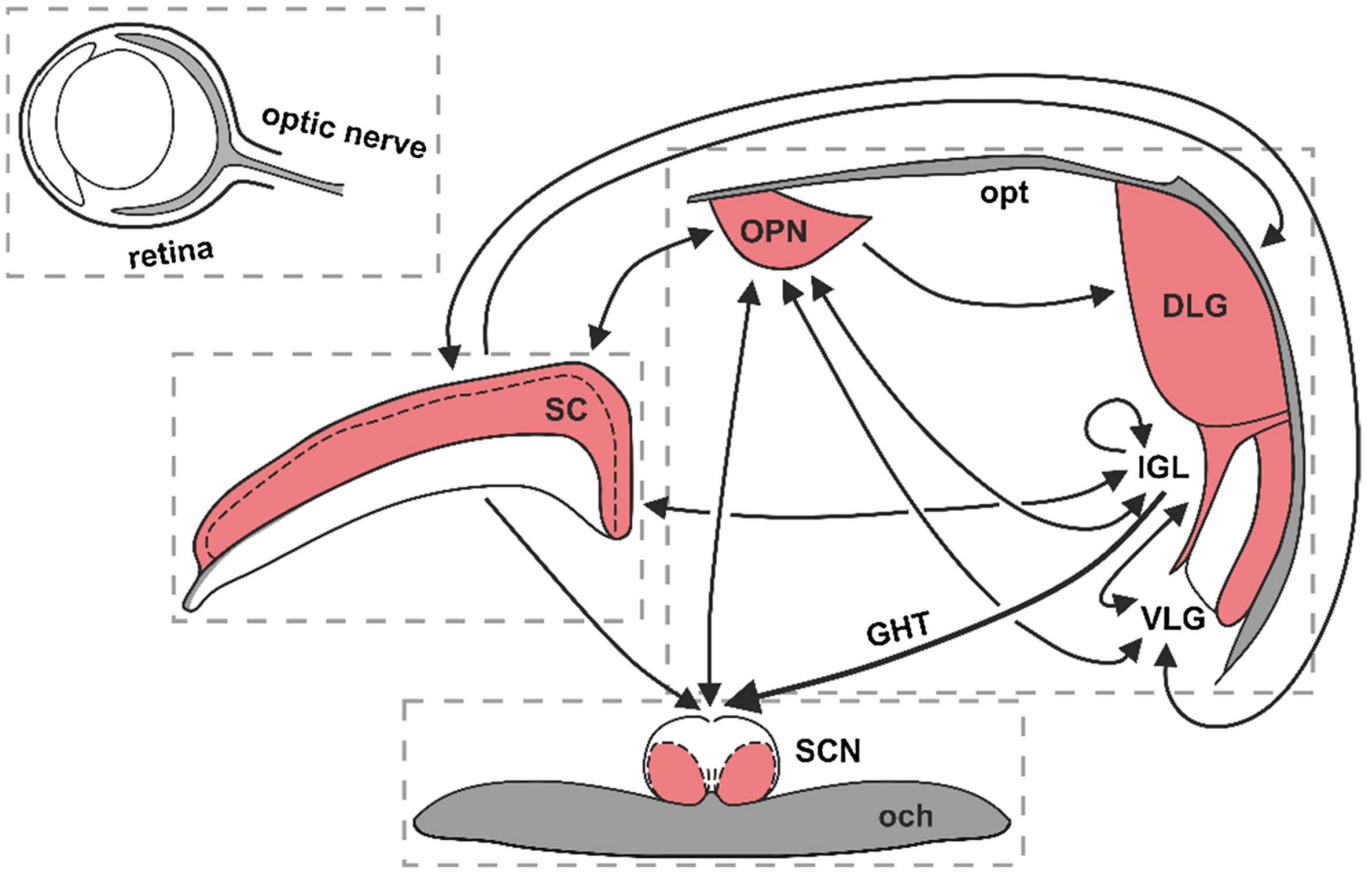
The functional neuroanatomy of the subcortical visual system (SVS). Red colouring denotes retinorecipient areas of the SVS. Black lines show neuronal connections among SVS nuclei, with arrows indicating the direction of connectivity. SC, superior colliculus; OPN, olivary pretectal nucleus; SCN, suprachiasmatic nucleus of the hypothalamus; DLG, dorsolateral geniculate; IGL, intergeniculate leaflet; VLG, ventrolateral geniculate; opt, optic tract; GHT, geniculo-hypothalamic tract; och, optic chiasm.

The subcortical visual system (SVS) is a collection of neuronal circuits in the midbrain, thalamus and hypothalamus that include the suprachiasmatic nucleus (SCN) of the hypothalamus, olivary pretectal nucleus (OPN), midbrain superior colliculus (SC) and thalamic lateral geniculate nucleus (LGN), among others. The LGN can be further divided into three anatomically and functionally distinct parts: the dorsolateral geniculate (DLG), ventrolateral geniculate (VLG) and the intergeniculate leaflet (IGL; Figure 1). These structures receive dense innervation both from the classical retinal ganglion cells (RGCs) and the intrinsically photosensitive RGCs (ipRGCs; Beier et al., 2021). The RGCs and ipRGCs integrate information from the rod and cone photoreceptors. In addition, ipRGCs express the photopigment melanopsin, which enables them to detect ambient light. These cells predominately communicate non-image-forming visual information to the brain for the pupillary light reflex, oculomotor functions and entrainment of circadian rhythms (Hattar et al., 2002; Schmidt et al., 2011). A subset of SVS neurons, such as the SCN and IGL, receives photic signals primarily from the ipRGCs (Morin and Allen, 2006; Schmidt et al., 2011).

**Figure 2:**
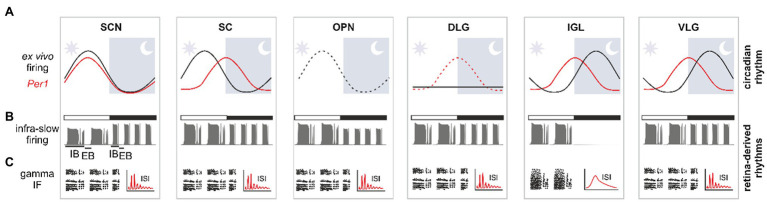
Multi-frequency organisation of oscillatory signals in the SVS. **(A)** Black and red sinewaves, respectively, representing the oscillatory relationship between spontaneous firing and *Per1* expression over a 24-h period *ex vivo*. The dotted black line in the olivary pretectal nucleus (OPN) is an extrapolation based on two daily time points. The horizontal black line in the dorsolateral geniculate nucleus (DLG) demonstrates a lack of daily rhythms in neuronal activity *ex vivo*. The red sinewave represents rhythmic core clock gene *Per1* expression in the SVS areas, with a dotted red line in the DLG coding its rhythmicity *in vivo* only. **(B)** Infra-slow rhythm is modulated by ambient light conditions. White bar shows light-adapted conditions, whereas black bar – dark-adapted recording. Note the enhanced frequency in darkness for most SVS areas, or a total silencing in the intergeniculate leaflet (IGL). **(C)** Gamma oscillation presented as the notable bands in the instantaneous frequency (IF) plots and as a multimodal inter-spike interval (ISI) histogram. The IGL stands an exception, with no gamma oscillation recorded in this structure. IB, intra-burst; EB, extra-burst phase; SCN, suprachiasmatic nucleus; SC, superior colliculus.

Subcortical visual system neuronal centres also share another common characteristic – they all display oscillatory activities in a variety of frequency bands (from the infra-slow <0.01Hz to faster 30–70Hz oscillations; Figure 2). Interestingly, aspects of these rhythms are highly synchronised across the SVS due to their common extrinsic origin from the eyes, but synchronicity across these structures is also generated intrinsically. Thus, it is useful to categorise these oscillators according to the source of their rhythmicity. An ‘autonomous oscillator’ intrinsically generates rhythmic changes in its electrical and/or molecular clock activity. This ability is propelled at single cell levels which generate activity that are highly synchronised, or phase-locked. This in turn ensures a robust rhythm output emanating from the whole structure. A ‘semi-autonomous oscillator’ possesses all the necessary neurophysiological processes to express an intrinsic rhythm. However, it requires occasional rhythmic entrainment inputs to maintain its phase at the whole circuit level, an inherent property of the poor interconnectivity among its neurons. By contrast, a ‘slave oscillator’ does not exhibit intrinsic oscillatory activity but its rhythmicity can be completely driven by an autonomous or semi-autonomous oscillator (Guilding and Piggins, 2007).

In mechanics, the term ‘coupling’ describes a connection between two oscillating systems which affects the oscillatory pattern of both systems. In neuronal systems where signal multiplexing is common, the term coupling is used as an umbrella concept to overtly describe the intricate functional connections between neurons and/or neuronal ensembles. The neurophysiology of coupling is diverse and complex, representing the mechanistic nature of connectivity *via* synaptic and/or diffusive pathways that transmit the various temporal features of neuronal excitability. Coupling between neuronal centres organises their firing patterns according to a certain oscillatory frequency (Buzsáki, 2006; Buzsáki and Llinás, 2017). Such organisation may be achieved by: (1) the coupling of multiple oscillators firing in the same frequency band (synchronisation) or (2) the coupling of different frequency bands embedded in the activity of a single oscillator (multiplexing).

## The Subcortical Visual System

The SCN, canonically considered as the master circadian clock, is a component of the SVS and is localised in the anterior hypothalamus just above the optic chiasm (Takahashi, 2017; Hastings et al., 2019). It can be subdivided into two parts: the dorsomedial (dSCN) or shell and ventrolateral SCN (vSCN) or core. The SCN is directly innervated by the retina and mostly by ipRGCs (Hattar et al., 2006; Schmidt et al., 2011; Lokshin et al., 2015; Fernandez et al., 2016; Chew et al., 2017; Hastings et al., 2019).

The OPN is another target of dense innervation from ipRGCs, mainly segregated to its shell, rather than the core region. The OPN is localised in the pretectal complex and, through its connections with the Edinger-Westphal nucleus, controls pupil dilation and relaxation. Light potently excites OPN neurons leading to pupil constriction; thus, the OPN is mainly responsible for the pupillary light reflex (Young and Lund, 1998; Hattar et al., 2006).

The SC is a multi-layered structure localised in the midbrain. Its superficial layers are retinorecipient, innervated predominately by the classical RGCs, thus constituting a subcortical visual information-processing centre. Its function in rodents is to orient the animal towards the visual stimuli by combining visual sensory processing with motor coordination (May, 2006; Cang et al., 2018; Beier et al., 2021).

The DLG is a vision-forming part of the LGN due to its direct connections with the primary visual cortex *via* thalamo-cortical neurons. It is the most distinct part of the LGN complex both from developmental and functional perspectives (Sefton et al., 2015). The VLG makes reciprocal connection with the SC and the cerebellum; thus, it is believed to play a role in eye movements and other oculomotor functions (Graybiel, 1974; Born and Schmidt, 2008). The VLG is further subdivided into the directly retinorecipient lateral lamina (VLGl) and the brainstem information-processing medial division (VLGm; Harrington, 1997; Kolmac and Mitrofanis, 2000).

The IGL is a relatively small but important structure, intercalated between the DLG and VLG. It serves as a non-image-forming visual brain region that transmits both photic and non-photic entrainment signals to the circadian clock in the SCN. The IGL has also been implicated in the photic regulation of mood and the sleep/wake cycle (Harrington, 1997; Huang et al., 2019; Shi et al., 2019; Fernandez et al., 2020). The LGN complex constitutes a retinorecipient thalamic hub that integrates photic and non-photic cues for image- and non-image-forming visual purposes (Monavarfeshani et al., 2017; Figure 1).

## Retinal Oscillations in the Gamma and Infra-Slow Frequency Bands

Undoubtedly, the linking feature of the otherwise diversified functions of the SVS is the dense retinal inputs (Beier et al., 2021). Despite the segregation of RGCs innervation to these different brain centres, there are striking similarities in their spontaneous firing patterns. The retina itself is a robust autonomous oscillator (Freeman et al., 2008; Guido et al., 2010; McMahon et al., 2014). The firing pattern of the RGCs is reflected in the rhythmic release of neurotransmitters from RGC terminals in the brain. This results in rhythmic patterns of predominately excitatory postsynaptic potential (EPSP) activity in targeted SVS neurons which provides rhythmic excitation to this neuronal system.

### Gamma Oscillation

The retinal network can oscillate in the gamma frequency range (also called the fast narrowband oscillation; Freeman et al., 2008; Guido et al., 2010; McMahon et al., 2014). These oscillations are thought to originate from single retinal cells and constitute a conserved feature across many species. Fast oscillations [with a frequency of 60–70Hz in freely moving and 30–40Hz in anaesthetised animals (Storchi et al., 2017)] are dependent on rods and cones inputs, but are generated by amacrine cells (Roy et al., 2017; Orlowska-Feuer et al., 2021). Studies in a number of species, including mice (Saleem et al., 2017; Storchi et al., 2017; Orlowska-Feuer et al., 2021), rats (Tsuji et al., 2016; Chrobok et al., 2018), cats (Heiss and Bornschein, 1966) and frogs (Ishikane et al., 1999), have shown that this oscillation frequency is transmitted to the SVS structures by the retina, including the VLG, OPN (Chrobok et al., 2018; Orlowska-Feuer et al., 2021), SCN (Tsuji et al., 2016), SC (Drwiega and Blasiak, unpublished observations) and finally the DLG (Storchi et al., 2017; Chrobok et al., 2018; Orlowska-Feuer et al., 2021; Figure 2). This fast oscillation has been hypothesised to play a role in image-forming vision, as the DLG gamma rhythm is further transmitted to the primary visual cortex (Saleem et al., 2017). Interestingly, the IGL was found not to organise its neuronal activity in the fast oscillatory band (Chrobok et al., 2018), presumably due to innervation from a distinct class of RGCs that do not oscillate in the gamma frequency band. This suggests an organised division of retinal information reaching distinct SVS structures. Due to a common retinal source, the fast oscillations are highly synchronised among neurons across the whole SVS structures. However, the phases of these oscillations are shifted between the LGN and OPN only by a few milliseconds, and this phase difference can be explained by the lag time of action potential trains reaching the various SVS nuclei from the retina (Chrobok et al., 2018). Recent findings have allowed us to form credible hypotheses on the potential role of the fast narrowband oscillations in the SVS. For example, similar to other fast rhythms found in the cortex, gamma oscillations may enhance the inter-area communication and facilitate the flow of sensory signal (Fries, 2009). As their amplitude and frequency track transient changes in irradiance, it is also possible that fast oscillations carry information about the features of the visual stimulus (Storchi et al., 2017; Chrobok et al., 2018). However, in non-image-forming SVS centres, including the SCN, the exact role for this oscillatory band remains elusive.

### Infra-Slow Rhythm

The other well-described retinal rhythm which is reflected in the neuronal activity of the SVS occurs in the infra-slow frequency band (<0.01Hz). Several research groups have recorded this oscillation within the SVS structures which encompasses all three parts of the LGN (Lewandowski and Błasiak, 2004; Filippov and Frolov, 2005; Blasiak and Lewandowski, 2013; Chrobok et al., 2018; Orlowska-Feuer et al., 2021), the SCN (Miller and Fuller, 1992; Tsuji et al., 2016), OPN (Szkudlarek et al., 2008, 2012; Chrobok et al., 2018; Orlowska-Feuer et al., 2021) and SC (Drwiega and Blasiak, unpublished observations; Figure 2). Due to its presence in the OPN, infra-slow oscillation is also measured in rhythmic changes of pupil size (Blasiak et al., 2013). However, much less is known of the function of this oscillation frequency in the SVS. A hint for a possible role comes from the observation that the frequency of infra-slow oscillation in the SVS is higher in animals when kept in darkness compared to when maintained under bright light conditions. This suggests that these oscillations may be involved in encoding ambient lighting conditions (Chrobok et al., 2018). However, to the best of our knowledge, there has not been any investigation as yet on whether the period of infra-slow oscillation reflects ambient light changes on a range of intensities. It is noteworthy that in most animal species studied thus far, this oscillation activity remains uninterrupted by darkness (Tsuji et al., 2016;Chrobok et al., 2018; Orlowska-Feuer et al., 2021), indicating that its spontaneous generation in the retina could be preserved in the absence of light. The IGL is the only known SVS structure to fall silent in darkness (Blasiak and Lewandowski, 2013; Chrobok et al., 2017, 2018, 2019); the infra-slow rhythm is eliminated at least at the level of neuronal firing. This exception is thought to occur due to the strong inhibitory network of the IGL; its retinorecipient cells do not sustain neuronal activity in the absence of strong retinal inputs.

### Gamma and Infra-Slow Activity Multiplexing

The concept that lower frequency rhythms modulate the amplitude of faster oscillations was unequivocally demonstrated in the hippocampus, where slower theta oscillations were found to amplify the amplitude of faster gamma rhythms (Jensen and Colgin, 2007). This was reflected as an increase in amplitude of the fast oscillations at the acrophase of the slower rhythm; and vice versa – a decrease or diminution in amplitude of the fast oscillation at the nadir of a slower rhythm. Similarly, in the SVS, infra-slow oscillations were found to gate the amplitude of gamma rhythms (Chrobok et al., 2018; Orlowska-Feuer et al., 2021). This is possible due to a bimodal distribution in firing rate at the intra-slow firing rhythm. During the intra-burst phase of the infra-slow rhythm (typically lasting about 1min; Figure 2), neuronal activity is high, and therefore, the gamma patterning of action potential firing can be maintained throughout this phase. Conversely, during the extra-burst (a period of neuronal silence; Figure 2), the retinal gamma activity is ‘gated.’ As both oscillatory patterns are generated by the retina, it is possible that aspects of the gamma activity gating observed in the SVS originate at the level of retina. However, emerging evidence suggests that gating is more likely to take place at the level of the SVS by extrinsic influence, such as signals coming from the brain stem (Chrobok et al., 2018). Remarkably, ambient light conditions in general do not influence the gating mechanism (Chrobok et al., 2018). It remains to be shown if rhythmic SVS cells form mutual connections with one another or inter-SVS connections are also maintained by non-oscillatory neurons. This would reveal if the SVS areas are synchronised by the retina alone and/or through the exchange of phasic information across the system.

A long-standing question in the field is at what stage of visual processing does the integration of information from the retinas occur, as each eye encodes distinct information on ambient light intensity. Recent study suggests that the IGL plays a key role in this integration process (Pienaar et al., 2018). As the retinas of both eyes are not directly connected, they act as two independent oscillators, autonomously driving both fast and infra-slow rhythms in the targeted tissues they innervate. Biological oscillations are not perfect sine waves, and two oscillations sharing the same frequency generated by two different sources do not necessarily correlate with each other. Our study has shown that retinal-driven rhythms in structures that are bilaterally localised in the brain hemispheres (e.g., in two LGNs) exhibit strikingly similar frequencies, but these rhythms are not phase-locked. In the rare event of a unilateral innervation of a single retinorecipient cell, oscillations in both frequency bands are not synchronised with neighbouring neurons innervated by the contralateral retina (Chrobok et al., 2018). The function and relevance of phase-locking remains yet to be understood.

## Modulation of Svs Neuronal Activity By Rhythmic Changes in General Brain State

The thalamus, including the LGN, is under a robust modulatory tone both from the cortical areas and arousing centres of the brainstem (Erisir et al., 1997; Harrington, 1997; Kolmac and Mitrofanis, 2000; Blasiak et al., 2006). Neuronal activities in the cortices and many brainstem nuclei exhibit profound changes that reflect general brain states during wakefulness and sleep (such as cortical activation and deactivation), which can be monitored by electrocorticogram (ECoG). In experimental setup, these general brain states can be studied in animals under urethane anaesthesia. During ‘urethane sleep,’ rodent ECoG exhibits rhythmic fluctuations which partially mimic the cyclically changing phases of sleep. During rapid eye movement (REM) or ‘cortical activation’, lower amplitude theta frequency waves are seen in the ECoG. In contrast, during non-rapid eye movement (NREM) or ‘cortical deactivation’ higher amplitude delta frequency waves occur in the ECoG (Balatoni and Détári, 2003; Clement et al., 2008; Pagliardini et al., 2013; Zhurakovskaya et al., 2016; Walczak and Błasiak, 2017).

**Figure 3:**
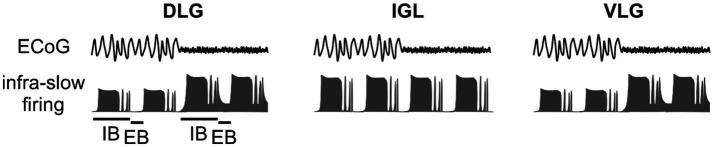
Modulation of infra-slow and gamma oscillations in the lateral geniculate nucleus (LGN) by the general brain state. Simultaneous recording of the electrocorticogram (ECoG) and neuronal activity in the thalamic SVS shows an impact of general brain state on the infra-slow oscillatory pattern. High amplitude, low frequency delta rhythm in the ECoG represents ‘cortical deactivation’. Contrary, low amplitude, high frequency theta oscillation is evident in the ECoG during ‘cortical activation’. Note that general brain state enhances neuronal firing in the DLG and ventrolateral geniculate nucleus (VLG) during both the intra-burst (IB) and extra-burst (EB) phase of the infra-slow oscillation. IGL, intergeniculate leaflet.

**Figure 4:**
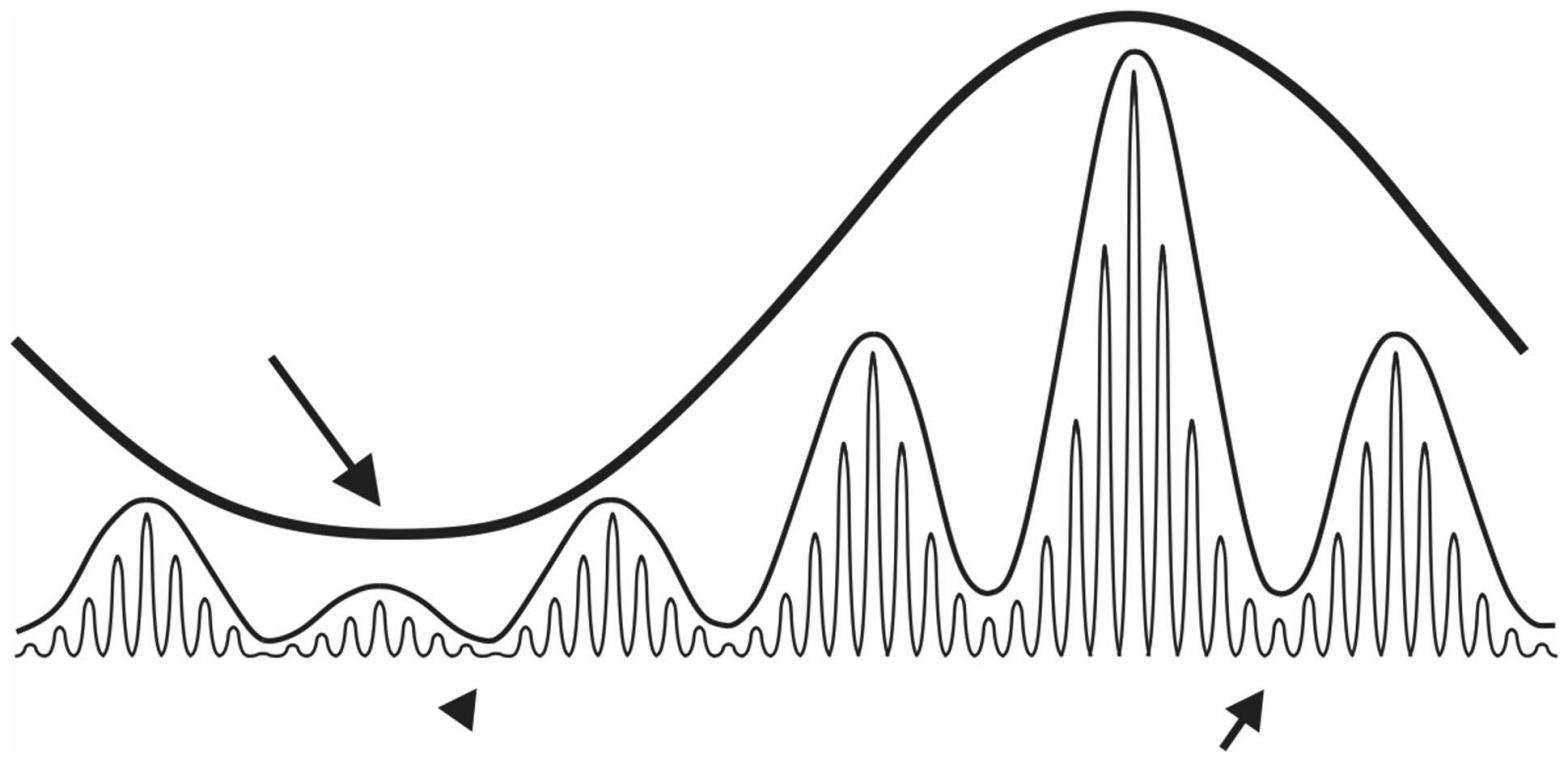
Schematic representation of oscillation multiplexing. The long black arrow indicates the temporal window where the slowest rhythm exhibits maximal amplitude suppression of the medium-frequency oscillation (maximal gating). Conversely, the amplitude of the medium-frequency oscillation is at its highest during the slow rhythm acrophase. Similarly, the medium-frequency oscillation modulates the amplitude of the fastest oscillation. Complete gating of the fastest oscillation occurs where the nadir of both slow and medium-frequency rhythms coincides (black arrowhead). Note, that gating of the fastest oscillation by the medium-frequency rhythm is not that efficient near the acrophase of the slow rhythm (short arrow).

Neuronal activity in the DLG and VLG, but not the IGL, is heavily modulated by these general brain states (Chrobok et al., 2018; Jeczmien-Lazur et al., 2019; Figure 3). It is most likely that brain state alterations that arise from sleep to wakefulness are the most profound changes to occur across a circadian cycle. It is therefore relevant to discuss how these general brain states modulate neurons exhibiting infra-slow and gamma oscillations. Recent evidence suggests that in the ‘cortical deactivation’ state, the extra-burst of the infra-slow oscillation is most often characterised by neuronal silencing (Chrobok et al., 2018). This means that while the brain is generally deactivated, the gamma rhythm is partially filtered at the level of the thalamus and may be transmitted to thalamic neuronal targets only during high activity in the intra-burst phase. The situation changes during the ‘cortical activation,’ which dramatically increases neuronal activity in the LGN (Chrobok et al., 2018). Thus, during the extra-burst, infra-slow oscillatory neurons remain active (although less active compared to intra-bursts; Figure 4). This implies that general brain state changes the gating properties of the thalamic neurons, and cortical activation promotes a constant flow of gamma oscillation [e.g., to the cortex (Saleem et al., 2017)], which amplitude is shaped by the infra-slow rhythm.

## Circadian Rhythmicity in the Subcortical Visual System

The SCN has traditionally been considered as the master circadian clock, which generates and coordinates daily rhythms in physiology across the brain and body (Takahashi, 2017; Hastings et al., 2019). With the discovery of multiple extra-SCN circadian timekeeping centres in the brain and peripheral tissues, it has now become clear that at least part of the rhythmic control of physiological processes must also develop at a tissue-specific local clock level (Guilding and Piggins, 2007; Albrecht, 2012; Myung et al., 2018; Begemann et al., 2020; Paul et al., 2020).

In support, recent investigations from our lab have reported circadian rhythmicity in core clock gene expression and neuronal activity in the SVS. This includes circadian oscillations in the IGL, VLG and SC (Chrobok et al., 2021b,c). Indeed, these rhythms in the electrical activity and clock gene expression were recorded under culture condition for several days in isolation from the SCN. This suggests that the circadian clock in the IGL, VLG and SC produces rhythms that are autonomous. In contrast, when isolated from SCN and in *ex vivo* conditions, the DLG shows circadian rhythmicity that quickly dampens and vanishes, suggesting that this brain locus functions as a slave oscillator. Additionally, our recent study reported a day/night difference in spontaneous firing rates in the OPN *ex vivo* (Chrobok et al., 2021a). Interestingly, the daily changes in neuronal activity are not in the same phase across the different SVS structures. For example, neuronal firing rate in the SCN, OPN and SC peaks during the day (light phase), whereas in the IGL and VLG, the firing peaks at night (dark phase; Belle et al., 2009; Hastings et al., 2018; Chrobok et al., 2021a,b,c; Figure 2). In the SCN, peak firing activity and *Per1* gene expression coincide (Takahashi, 2017), whereas this is not the case for the IGL, SC and VLG (Figure 2). This may suggest a differential clock-controlled regulation of firing activity in these SVS structures which could be important for tissue-specific timekeeping across the SVS.

How circadian rhythms in the firing rate affect infra-slow and gamma oscillations remain an open question (Figure 4). A recent *in vivo* study has reported more frequent infra-slow oscillations in the SCN during the subjective day (when the SCN firing rate is high), than the subjective night (Tsuji et al., 2016). This may imply a circadian gating of infra-slow oscillations in this structure, similar to the gating effects of theta oscillations on gamma rhythms in the hippocampus (Jensen and Colgin, 2007). It is also plausible that this measured day-night change in SCN infra-slow oscillation frequency may be due to the inability for these investigators to reliably assess firing pattern in the SCN at night, when SCN neurons are hyperpolarised and firing at significantly reduced rate (Belle et al., 2009; Hastings et al., 2018). The possibility for circadian gating in the LGN would also be important. During the behaviourally active dark phase when photic information is sparse but critical for exploratory behaviour, the circadian drive of LGN neuronal activity peaks. This peaking in LGN neuronal excitability may serve to bolster information-carrying infra-slow and gamma oscillations across the SVS.

## Concluding Remarks

The SVS is a unique network of interconnected midbrain, thalamic and hypothalamic brain structures that operates in concert to process environmental light conditions. Its overall neuronal activity rhythm emerges from a combination of intrinsic cellular properties shaped by external oscillatory inputs, such as the electrical signal from the retina. Several oscillation frequencies are detected in the SVS areas, including gamma, infra-slow and circadian rhythms which may shape its operations. In addition, global brain states can also influence activity in the SVS. The plethora of frequency bands that emerge in the SVS, through the coupling of single SVS neurons, synchronise, interfere and multiplex with one another. Such complex interactions provide opportunities for slow rhythms to gate oscillations occurring at faster frequencies (Belle and Diekman, 2018; Figure 4). We hypothesise that gating created by multiscale coupling enables the SVS to manage important aspects of visual information flow in the brain at appropriate time. Unravelling the functional role for these distinct rhythms is challenging but critical if we are to understand the working brain.

## Data Availability Statement

The original contributions presented in the study are included in the article/supplementary material, further inquiries can be directed to the corresponding authors.

## Author Contributions

LC wrote the first version of the manuscript and prepared illustrations. MB and JM substantially edited and revised the manuscript. All authors contributed to the article and approved the submitted version.

## Funding

This work is funded by a National Science Centre grant ‘Sonatina 2’ (2018/28/C/NZ4/00099) to LC, Biotechnology and Biological Sciences Research Council (BBSRC) Award to MDCB (BB/S01764X/1), and the Higher Education Sprout Project by the Taiwan Ministry of Education (DP2-109-21121-01-N-01, DP2-110-21121-01-N-01), the Taiwan Ministry of Science and Technology (110-2311-B-038-003, 110-2314-B-038-162, 110-2314-B-006-113, 109-2320-B-038-020, 109-2314-B-038-071, 109-2314-B-038-106-MY3) and Taipei Medical University (TMU107-AE1-B15, 107TMU-SHH-03) to JM.

## Conflict of Interest

The authors declare that the research was conducted in the absence of any commercial or financial relationships that could be construed as a potential conflict of interest.

## Publisher’s Note

All claims expressed in this article are solely those of the authors and do not necessarily represent those of their affiliated organizations, or those of the publisher, the editors and the reviewers. Any product that may be evaluated in this article, or claim that may be made by its manufacturer, is not guaranteed or endorsed by the publisher.
